# Air Duster Inhalant Abuse Causing Non-ST Elevation Myocardial Infarction

**DOI:** 10.7759/cureus.8402

**Published:** 2020-06-01

**Authors:** Shiliang A Cao, Madhab Ray, Nikolai Klebanov

**Affiliations:** 1 Anesthesiology, Massachusetts General Hospital, Boston, USA; 2 Internal Medicine, Signature Healthcare Brockton Hospital, Brockton, USA; 3 Anesthesiology, Harvard Medical School, Boston, USA; 4 Dermatology, Massachusetts General Hospital, Boston, USA; 5 Dermatology, Harvard Medical School, Boston, USA

**Keywords:** inhalant abuse, huffing, duster, fluorinated hydrocarbon, hydrocarbon, hydrocarbon toxicity, air duster, nstemi

## Abstract

Inhalant abuse, also known as huffing, is common among teenagers and adolescents in the United States and worldwide. Inhaled aerosols are dangerous due to both the presence of volatile hydrocarbons causing direct organ damage and the risk of the compressed air causing physical trauma (e.g. expansion, barotrauma) or skin trauma from chemical or temperature burn. Here, we present the case of a 35-year-old man who was inhaling multiple canisters of Dust-Off (Falcon Safety Products Inc., Branchburg, NJ) keyboard air duster daily for approximately one month. He presented with intermittent burning chest pains, and was found to have elevated troponin (peak 17 ng/mL, normal range 0-0.5 ng/mL) without ST-segment elevations, concerning for non-ST elevation myocardial infarction (NSTEMI) as well as elevated aminotransferases and elevated serum creatinine. He was treated conservatively with supportive measures, with successful resolution of his laboratory abnormalities as well as his chest pain. Clinicians should be aware of the possible medical complications of inhalant abuse, and the expected clinical course. In this case, we aim to demonstrate the acute onset and self-resolution of significant cardiomyocyte damage in a young male patient abusing duster.

## Introduction

Inhalants are volatile products, usually aerosols, that are typically easily available, inexpensive, and able to rapidly induce euphoria. Examples of ingredients found in these inhalants include aromatic hydrocarbons, nitrous oxide, and volatile alkyl nitrites. Inhalant abuse, also known as huffing, is common among teenagers and adolescents, but can occur in any age group; according to the National Institute on Drug Abuse, nearly 21.7 million Americans aged 12 years and older have used inhalants at least once in their lives [1]. Huffing can cause symptoms such as respiratory distress, hypoxia, nausea, vomiting, central nervous system depression, and myocardial sensitization [2].

Dust-Off (Falcon Safety Products Inc., Branchburg, NJ), one particular type of such an inhalant, is a propellant cleaner meant to remove dust and debris from keyboards, screens, and other electronics. Its active ingredient is difluoroethane, a colorless, liquefied hydrocarbon gas. The material safety data sheet for Dust-Off reports that inhaling high concentrations of this product may cause heart irregularities, unconsciousness, or death and that high exposures to this product may cause irritation of the nose, throat, and lungs with cough, difficulty breathing or shortness of breath, temporary alteration of the heart’s electrical activity with irregular pulse, palpitations, or inadequate circulation, or abnormal kidney function.

Here, we report a case of cardiac, hepatic, and renal toxicity in a young male patient abusing duster, who subsequently improved clinically with supportive therapy and was discharged. He planned to voluntarily present to a drug abuse rehabilitation facility upon discharge. It is important for physicians to be aware of the clinical signs of duster abuse to appropriately guide acute medical management.

## Case presentation

A 35-year-old Caucasian male patient with a history of depression and opioid use disorder presented to the emergency department with intermittent chest burning for one month. At the time of presentation, he had been clean from opioids for 28 months and currently on a buprenorphine (Suboxone) program. He admitted that approximately one month prior, he started buying air dusters from a local supermarket, as this was a way for him to feel intoxicated without a positive drug screen. He was inhaling the dusters every day, going through multiple 300 mL cans daily. In addition to the intermittent chest burning, he also reports three weeks of sharp episodic pain in the left flank worse with movement, generalized muscle weakness, myalgias, and complaint of intermittent headaches described as “warm” and significant nausea while huffing.

He reported his last use of the duster about one hour prior to emergency room admission. His vital signs included a heart rate of 112 beats/min, blood pressure of 126/90 mmHg, respiratory rate of 18 breaths/min, and oxygen saturation of 99% on room air. An initial electrocardiogram (EKG) was obtained which showed sinus tachycardia and no ST-segment elevations. Other notable laboratory findings included a leukocytosis to 15.2 K/µL (normal range 4-10 K/µL) with neutrophilic predominance, bicarbonate of 17 mEq/L (normal range 23-28 K/µL), anion gap of 17 mEq/L (normal range 3-10 mEq/L), blood urea nitrogen 25 mg/dL (normal range 8-20 mg/dL), creatinine 1.7 mg/dL (normal range 0.7-1.3 mg/dL), aspartate transaminase (AST) 68 units/L (normal range 0-35 units/L), alanine transaminase (ALT) 66 units/L (normal range 0-35 units/L), alkaline phosphatase 129 units/L (normal range 36-92 units/L), and creatine kinase 397 units/L (normal range 30-170 units/L). His initial troponin I in the emergency department was found to be elevated to 1.85 ng/mL (normal range 0-0.5 ng/mL). Urine drug screening was positive only for buprenorphine. A chest x-ray was obtained which had no significant abnormalities and was negative for infection.

**Figure 1:**
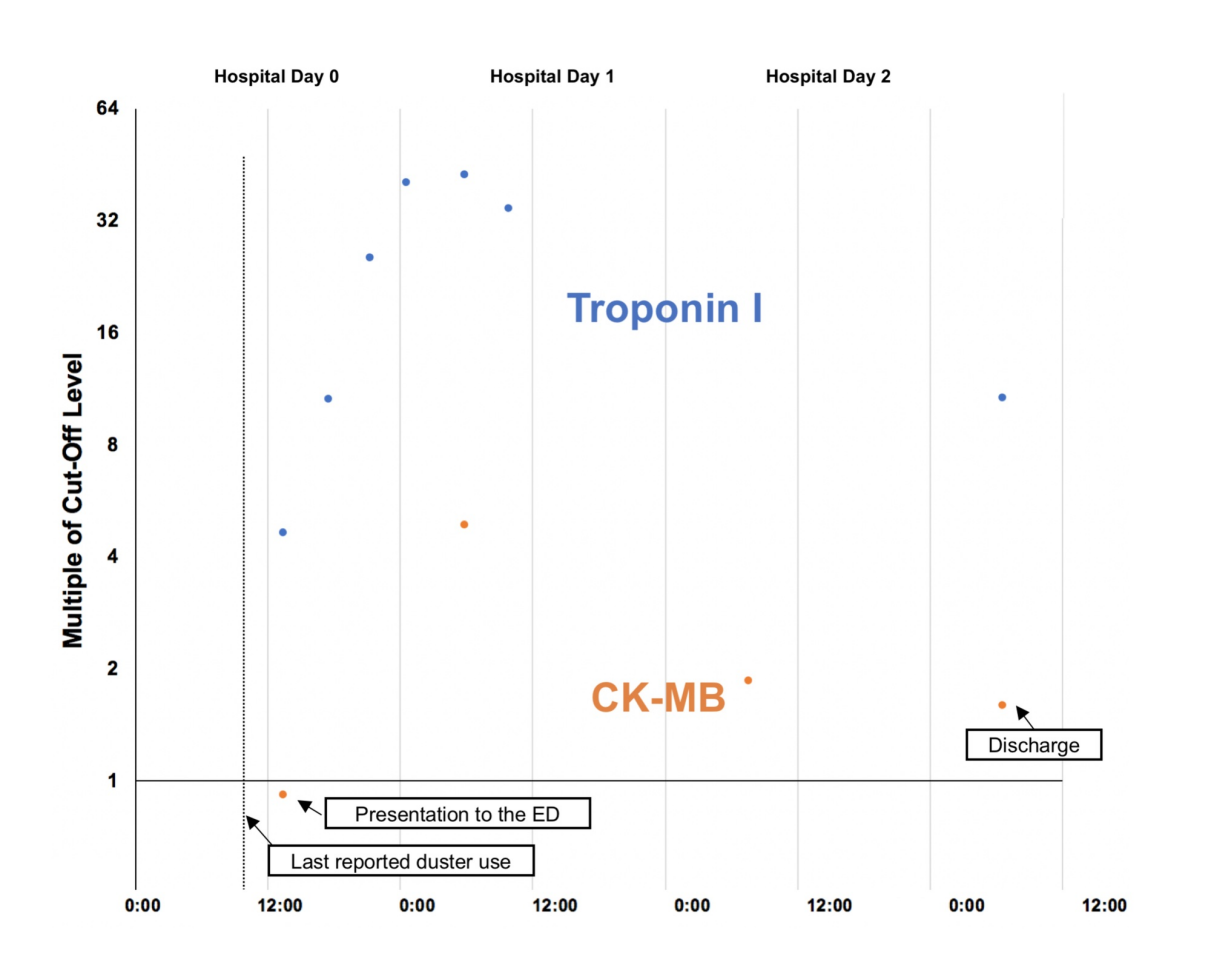
Troponin I and CK-MB levels measured starting around two to three hours following last reported duster use with associated burning chest pain. CK-MB: creatine kinase-MB

The patient was given aspirin 325 mg, 5 mg of IV metoprolol tartrate, and 1 L of normal saline, and was started on the hospital’s weight-based heparin infusion protocol for acute coronary syndrome. The patient’s troponin I levels rose dramatically from the time of arrival to the hospital at 1.85 ng/mL to peak at 17.01 ng/mL or 42.5 multiples of the cut-off (MOC) on the morning of hospital day 1. His creatine kinase-MB (CK-MB) also increased accordingly. At the time of arrival to the hospital, the CK-MB was 4.6 ng/mL (0.92 MOC, normal range 0-4.9 ng/mL), rising to 24.4 (4.88 MOC) on the morning of hospital day 1 (Figure 1). 

**Figure 2:**
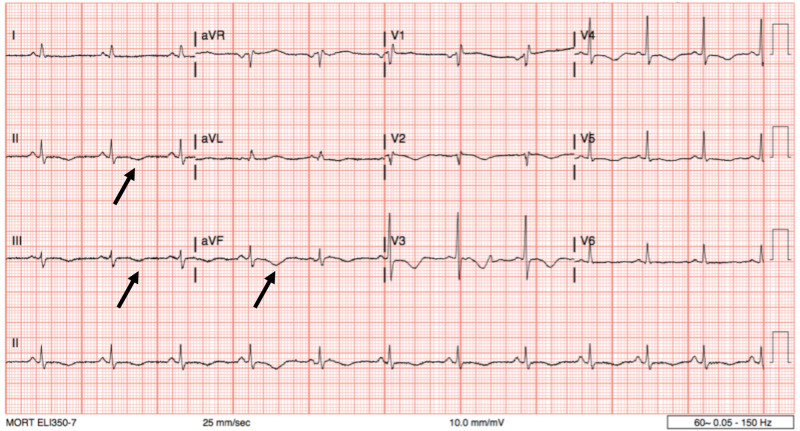
Electrocardiogram reveals T-wave inversions in the inferior leads (black arrows), QTc prolongation is also noted (QTc = 526 ms).

A repeat EKG was notable for sinus tachycardia, and prominent T-wave inversions in the inferior leads consistent with ischemia; the overall clinical picture was consistent with non-ST elevation myocardial infarction or NSTEMI (Figure 2).

Regional poison control was consulted, who recommended cardiac workup and supportive care. Per cardiology recommendations, the patient continued on a weight-based heparin protocol and was started on daily aspirin and daily statin medications. A transthoracic echocardiogram (TTE) showed an ejection fraction of 60% with normal left ventricular function. On hospital day 2, the patient underwent left heart catheterization, which showed clear coronary vessels. Clinically, the patient remained chest pain free throughout the admission. On discharge, his troponin I remained elevated at 4.3 ng/mL (10.7 MOC), and his CK-MB remained elevated at 8.0 ng/mL (1.6 MOC).

In addition to his primarily cardiac presentation, we found that the patient had likely mild hepatic and renal damage given his elevated transaminases and elevated plasma blood urea nitrogen (BUN) and creatinine. His AST increased from 68 to 102 units/L at peak, and ALT increased from 66 to 121 units/L peak. Hepatitis serologies were normal, and right upper quadrant ultrasound was unremarkable. His BUN and creatinine levels suggested intrinsic kidney injury. Renal ultrasound was unremarkable. His transaminase abnormalities and renal marker elevations resolved with supportive care.

## Discussion

Short-term inhalant abuse is not uncommon among adolescents in the United States. Per the National Survey on Drug Use and Health (NSDUH) in 2011, 9% of the United States population aged 12 years and older had been estimated to use an inhalant for its psychoactive properties at least once; however, most discontinue this quickly [3]. Given the significant medical toxicities associated with the hydrocarbons such as butane, propane, and chlorofluorocarbons contained in the inhalants, even short-term users are at risk of developing acute organ injuries secondary to inhalant use.

Several cases of huffing leading to elevated cardiac and liver enzymes have been reported in the literature. In one case, a 20-year-old man inhaled “Dust-off” spray two to three days prior to admission. His cardiac enzymes were also found to be elevated on admission, and a TTE showed global left ventricular dysfunction with an ejection fraction of 10%-15% [4]. Similar to our case, his cardiac enzymes trended downward, and subsequently his dysfunction spontaneously resolved.

In another case, a 34-year-old man was found down in a parking lot after huffing 15 cans of Dust-Off [2]. The patient experienced a torsades de pointes arrhythmia, and echocardiogram revealed left and right ventricular dysfunction with an ejection fraction of 25%; he had an elevated troponin that peaked at 20 ng/mL. This patient also had elevations in liver enzymes: AST 64 units/L with 17,432 units/L at peak (normal range 0-35 units/L), ALT 48 U/L (normal range 0-35 units/L), and creatine kinase 5,058 units/L at peak (normal range 30-170 units/L), which were more pronounced than those in our case.

We also found multiple reports of huffing abuse causing myocarditis, pneumopericardium, rapid airway compromise, and chemical or thermal burns [1,5-7]. Inhalant abuse should be considered as a differential diagnosis in young patients presenting with elevated cardiac, liver, or possibly renal markers of unknown etiology. Overall, given the risk of mechanical trauma from pressurized or rapidly cooled air and the medical risks associated with inhaling hydrocarbons and solvents, it is important for clinicians to be aware of the wide range of bodily injuries that could be associated with duster abuse.

Our patient presented with intermittent burning chest pain and significant elevation in his troponin I without initial EKG changes suggestive of acute ischemia. It is possible that his cardiac damage was due to both direct toxic effects of the hydrocarbons on the myocardium, and possibly demand ischemia due to transient hypoxia while inhaling the duster. His cardiac catheterization revealed clear vessels, which further increases the risk attributable to the duster in this particular case.

## Conclusions

This article highlights a case of cardiotoxicity and hepatotoxicity caused by duster abuse. A 35-year-old man who was inhaling dusters for one month presented with intermittent burning chest pains and was found to have troponin elevated to 17 ng/mL. He was started on aspirin, statin, beta-blocker, and weight-based heparin infusion, and improved with supportive care. Despite the rarity of these cases, inhalant intoxication should be considered as a differential diagnosis in patients with elevated cardiac and liver enzymes of unknown etiology. Reassuringly, our case suggests that patients with such laboratory abnormalities may improve in the short term with supportive care. Further research is needed to elucidate the pathophysiology of cardiotoxic and hepatotoxic effects of inhalants.
